# Changes in soil microbial communities at Jinsha earthen site are associated with earthen site deterioration

**DOI:** 10.1186/s12866-020-01836-1

**Published:** 2020-06-05

**Authors:** Jing Li, Xiaoyue Zhang, Lin Xiao, Ke Liu, Yue Li, Ziwei Zhang, Qiang Chen, Xiaolin Ao, Decong Liao, Yunfu Gu, Menggen Ma, Xiumei Yu, Quanju Xiang, Ji Chen, Xiaoping Zhang, Tao Yang, Petri Penttinen, Ke Zhao

**Affiliations:** 1 Department of Microbiology, College of Resources, Sichuan Agricultural University, Yaan, 625000 PR China; 2 Chengdu Institute of Cultural Relics and Archaeology, Chengdu, 610072 Sichuan China; 3 Jinsha site museum, Chengdu, Sichuan 610072 PR China

**Keywords:** Earthen site, Soil microbiome, Deterioration, High-throughput sequencing

## Abstract

**Background:**

Earthen sites are immobile cultural relics and an important part of cultural heritage with historical, artistic and scientific values. The deterioration of features in earthen sites result in permanent loss of cultural information, causing immeasurable damage to the study of history and culture. Most research on the deterioration of earthen sites has concentrated on physicochemical factors, and information on microbial communities in earthen sites and their relationship with the earthen site deterioration is scarce. We used high-throughput sequencing to analyze bacterial and fungal communities in soils from earthen walls with different degree of deterioration at Jinsha earthen site to characterize the microbial communities and their correlation with environmental factors, and to compare microbial community structures and the relative abundances of individual taxa associated with different degree of deterioration for identifying possible marker taxa.

**Results:**

The relative abundances of Proteobacteria and Firmicutes were higher and that of Actinobacteria lower with higher degree of deterioration. At the genus level, the relative abundances of *Rubrobacter* were highest in all sample groups except in the most deteriorated samples where that of *Bacteroides* was highest. The relative abundance of the yeast genus *Candida* was highest in the severely deteriorated sample group. The bacterial phylum Bacteroidetes and genus *Bacteroides*, and fungal class Saccharomycetes that includes *Candida* sp. were specific for the most deteriorated samples. For both bacteria and fungi, the differences in community composition were associated with differences in EC, moisture, pH, and the concentrations of NH_4_^+^, K^+^, Mg^2+^, Ca^2+^ and SO_4_^2−^.

**Conclusion:**

The microbial communities in soil with different degree of deterioration were distinctly different, and deterioration was accompanied with bigger changes in the bacterial than in the fungal community. In addition, the deteriorated soil contained higher concentrations of soluble salts. Potentially, the accumulation of *Bacteroides* and *Candida* plays an important role in the deterioration of earthen features. Further work is needed to conclude whether controlling the growth of the bacteria and fungi with high relative abundances in the deteriorated samples can be applied to alleviate deterioration.

## Background

Earthen sites are mainly soil formations produced by ancient activities. These immobile cultural relics are part of cultural heritage with historical, artistic and scientific values [1]. In China, the earthen sites with significant archaeological value include for example heritage sites along the Silk Road, the Great Wall remains and Beacon Tower in Gansu province [2, 3]. Preservation of these sites is essential for studying history and culture. However, for thousands of years, earthen sites have been subject to environmental impacts such as erosion due to severe winds and heavy rainfall, earthquakes, and fluctuation in temperature and humidity [3]. The deterioration of features in earthen sites include loose efflorescence, weathering, cracks, and collapses [4–6]. The deterioration, mediated by physical, chemical and biological processes [4–7], results in permanent loss of cultural information, causing immeasurable damage to studying history and culture.

Jinsha earthen site was discovered in Chengdu, Sichuan, in the southwest of China in February 2001. Jinsha, the capital of ancient Sun Kingdom that dates back to 12th to seventh century BCE (approximately 2900–3200 years ago), is considered as an ancient civilization center along Yangtze River. So far, archaeologists have unearthed important features of large-scale palace foundation, sacrificial area, residential area, and a burial site. On the site, numerous artifacts have been unearthed, including more than 5000 articles of gold, bronze, jade and stone, as well as millions of potsherds, tons of ivory and thousands of boar tusks and deer horns. The Jinsha Site Museum has built the Relics Hall in the excavation site for the protection, study and exhibition of Jinsha culture and ancient Shu civilization. Even though the Relics Hall has alleviated damage by external factors, such as wind, sun and rain, the earthen features in the in semi-open Relics Hall have suffered different degrees of deterioration, including salinization, efflorescence, and cracking.

Most research on the deterioration of earthen sites has concentrated on physicochemical factors [1, 2, 8–10]. Microorganisms play an important role in weathering of stone monuments [11], and probably cause serious damage to earthen sites as well. However, information on microbial communities in earthen sites and their relationship with the earthen site deterioration is scarce. We used high-throughput sequencing to analyze bacterial and fungal communities in soils from earthen walls with different degree of deterioration at Jinsha earthen site. The objectives were to (1) characterize the microbial communities and their correlation with environmental factors, and (2) compare the community structures and the relative abundances of individual taxa associated with different degree of deterioration for identifying possible marker taxa. We hypothesized that microbial community composition would vary depending on deterioration degree. The results were expected to pave way for means to alleviate the deterioration of earthen features.

## Results

### The physicochemical properties

The physicochemical properties of the soil samples varied with the deterioration degree of the earthen wall (Table 1). Moisture was higher in the moderately and severely deteriorated samples S3 and S4 than in the not or slightly deteriorated samples S1 and S2 (*p* > 0.05), and pH ranged from 7.32 in S4 to 7.48 in S1 (*p* > 0.05). EC value and the concentrations of Mg^2+^, Ca^2+^ and SO_4_^2−^ were higher in the more deteriorated samples (*P* < 0.05), whereas the concentrations of NH_4_^+^ and K^+^ were lower (*p* > 0.05). In the SEM-EDS spot analysis, the relative proportions of C, S, O and Mg elements were higher in samples S3 and S4 than in samples S1 and S2, and those of Al, Si and K were lower (*p* > 0.05) (Additional File 1: Table S1).

**Table 1:** Physicochemical properties of soil with different degree of deterioration from Jinsha earthen site

Sample	Moisture (%)	pH	EC	Contents of soluble salts (mmol kg^− 1^)							
			(μs cm^−1^)	Na^+^	NH_4_^+^	K^+^	Mg^2+^	Ca^2+^	Cl^−^	NO_3_^−^	SO_4_^2−^
S1	4.56± 0.11^b^	7.48± 0.04^a^	6.74± 0.05^d^	12.53 ± 3.66^b^	3.80± 0.02^a^	4.94± 0.04^a^	193.933± 0.99^d^	4.45± 0.08^d^	21.12 ± 0.19^c^	10.44 ± 0.46^b^	111.39± 0.91^d^
S2	4.69± 0.04^b^	7.41± 0.03^ab^	10.45± 0.40^c^	18.59 ± 0.02^a^	3.62± 0.02^b^	4.70± 0.02^b^	668.29± 1.44^c^	11.25± 0.02^c^	51.43 ± 0.56^a^	33.46 ± 0.42^a^	205.04± 0.92^c^
S3	4.84± 0.10^a^	7.35± 0.09^ab^	11.44± 0.67^b^	10.41 ± 0.05^c^	2.45± 0.04^c^	4.56± 0.02^c^	682.74± 2.19^b^	43.15± 0.33^b^	22.80 ± 0.79^b^	7.25± 0.16^c^	326.42± 2.24^b^
S4	4.98± 0.04^a^	7.32± 0.08^b^	13.92± 0.63^a^	10.40 ± 0.16^c^	0.0014± 0.0003^d^	4.55± 0.04^c^	1519.70± 1.56^a^	143.07± 0.94^a^	13.67 ± 0.30^d^	9.87± 0.08^b^	946.48± 2.12^a^

The results are average ± standard deviation (*n* = 3). Different superscript letters in a column indicate statistical significant difference (*p* < 0.05) in the least significant difference test. S1, no obvious deterioration; S2, mild deterioration; S3, moderate deterioration; S4, severe deterioration

### The diversity of microbial communities

The bacterial and fungal communities in the soil samples were analyzed using amplicon sequencing targeting the 16S rRNA gene and ITS, respectively. The 816,336 16S rRNA gene amplicons were divided into 2555 bacterial operational taxonomic units (OTUs) at ≥97% similarity. The Good’s coverage was above 99.7 and 99.9% for 16S rRNA gene and ITS amplicons, respectively, and all the rarefaction curves reached an asymptote, showing that the amplicons represented well the sampled populations (Figure S1). The average number of bacterial OTUs per sample group ranged from 553 to 718 (Table 2). The 957,322 ITS amplicons were divided into 899 fungal OTUs. The average number of fungal OTUs per sample group ranged from 249 to 355 (Table 2). For bacteria, richness was higher in S4 than in the other sample groups, and diversity was higher in S3 and S4 than in S1 and S2 (*p* < 0.05) (Table 2). For fungi, richness was higher in S1 than in S4 (*p* < 0.05) (Table 2), and diversities were on the same level in all the sample groups.

**Table 2:** Richness and diversity of bacterial and fungal communities in soil with different degree of deterioration

	Bacteria			Fungi		
Sample	OTUs	Chao1	Shannon	OTUs	Chao1	Shannon
S1	570 ± 76^b^	641 ± 70^b^	3.456 ± 0.25^b^	355 ± 26^a^	392 ± 15^a^	5.86 ± 0.05^a^
S2	553 ± 163^b^	598 ± 158^b^	3.76 ± 0.974^b^	288 ± 29^ab^	344 ± 61^ab^	4.603 ± 0.49^a^
S3	656 ± 128^b^	672 ± 128^b^	6.771 ± 0.046^a^	325 ± 18^ab^	361 ± 11^ab^	5.67 ± 0.088^a^
S4	718 ± 375^a^	741 ± 362^a^	6.649 ± 1.142^a^	249 ± 80^b^	264 ± 83^b^	4.255 ± 1.863^a^

The results are average ± standard deviation (*n* = 3). Different superscript letters in a column indicate statistical significant difference (*p* < 0.05) in the least significant difference test. S1, no obvious deterioration; S2, mild deterioration; S3, moderate deterioration; S4, severe deterioration

**Fig. 1:**
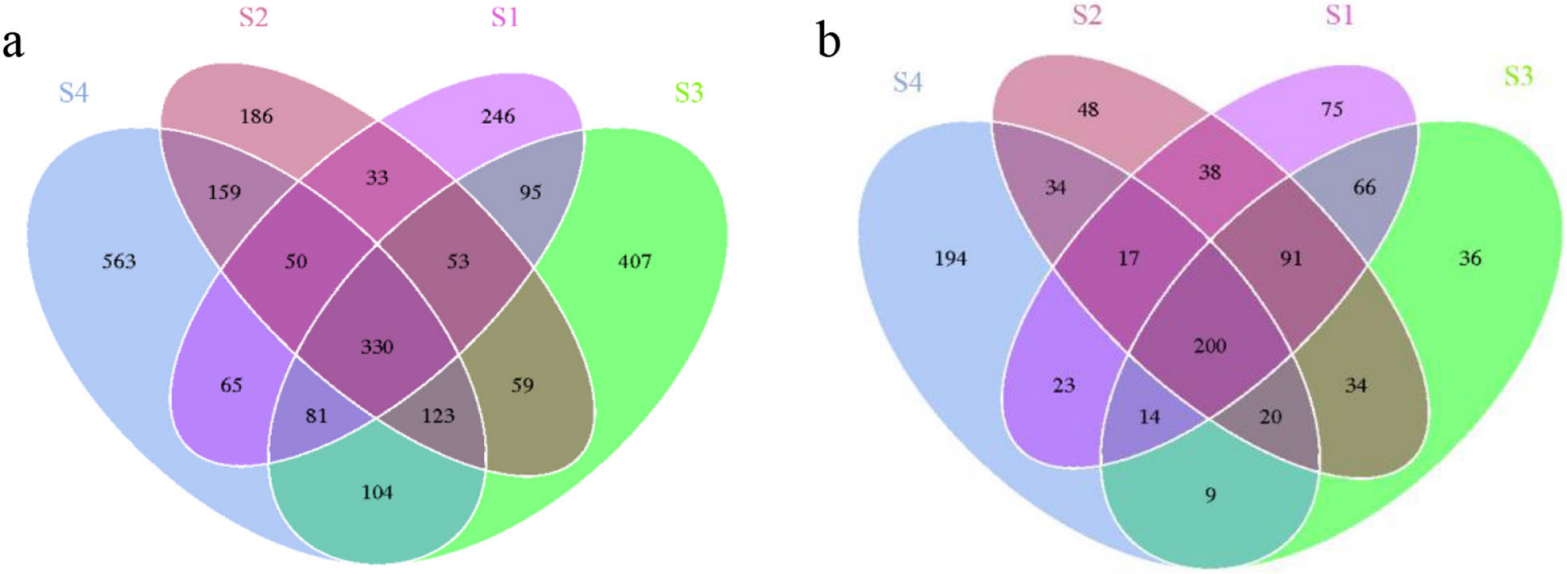
The unique and shared bacterial (**a**) and fungal (**b**) OTUs in soil with different degree of deterioration from Jinsha earthen site. S1, no obvious deterioration; S2, mild deterioration; S3, moderate deterioration; S4, severe deterioration

**Fig. 2:**
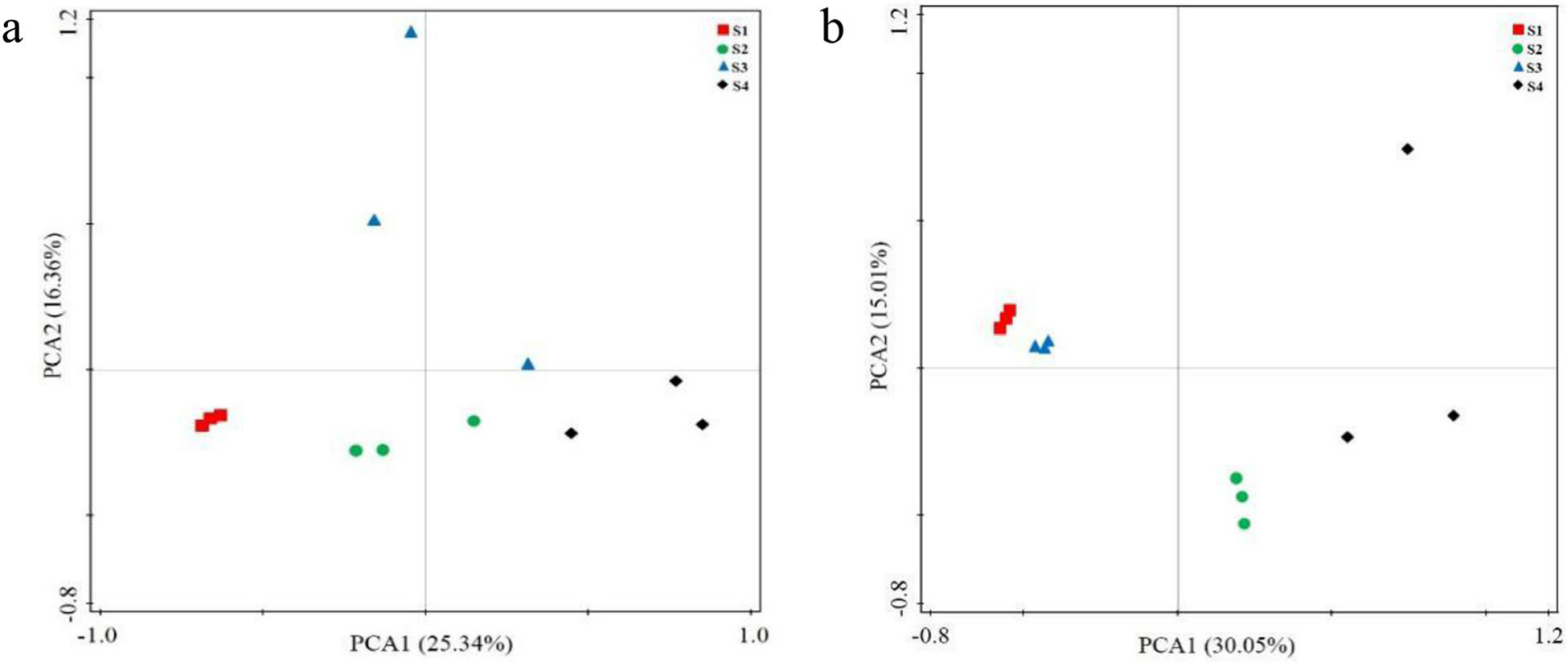
Principal component analysis (PCA) of bacterial (**a**) and fungal (**b**) communities in soil with different degree of deterioration from Jinsha earthen site. S1, no obvious deterioration; S2, mild deterioration; S3, moderate deterioration; S4, severe deterioration

Altogether 330 bacterial and 200 fungal OTUs were detected in all the four sample groups (Fig. 1). The highest number of unique bacterial OTUs was detected in S4 and the lowest in S2. The highest number of unique fungal OTUs was detected in S4 (Fig. 1). Both the bacterial and fungal communities in the not deteriorated and severely deteriorated sample groups S1 and S4, respectively, were clearly separated in the principal component analysis (PCA) (Fig. 2).

### Distribution of microbial community in sample groups

**Fig. 3:**
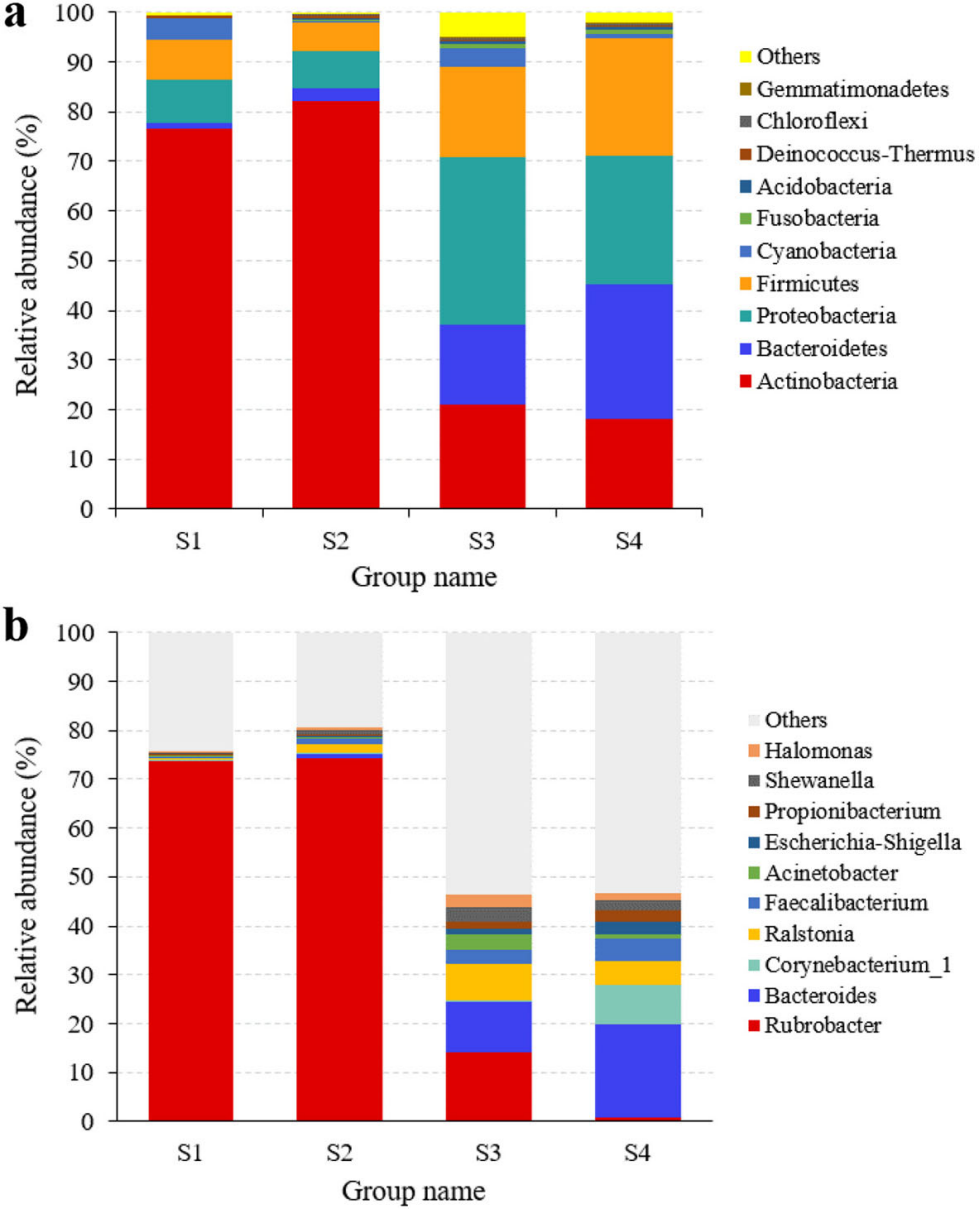
Relative abundances of bacterial phyla (**a**) and genera (**b**) l in soil with different degree of deterioration from Jinsha earthen site. S1, no obvious deterioration; S2, mild deterioration; S3, moderate deterioration; S4, severe deterioration

The bacterial OTUs were assigned into 36 phyla and 617 genera. Actinobacteria, Bacteroidetes, Proteobacteria and Firmicutes were the most abundant phyla (Fig. 3a). The relative abundances of Actinobacteria were highest in the sample groups S1 and S2, and those of Proteobacteria and Firmicutes in S3 and S4 (*p* < 0.05) (Additional File 1: Table S2).

At the genus level, the relative abundances of *Rubrobacter* were highest in all sample groups except S4 where that of *Bacteroides* was highest (Fig. 3b). Compared with the S1 and S2, the relative abundances of *Shewanella* were higher in S3 and S4 (*p* > 0.05). The results showed that the bacterial community compositions in sample groups with different degree of deterioration were significantly different (Additional File 1: Table S3).

**Fig. 4:**
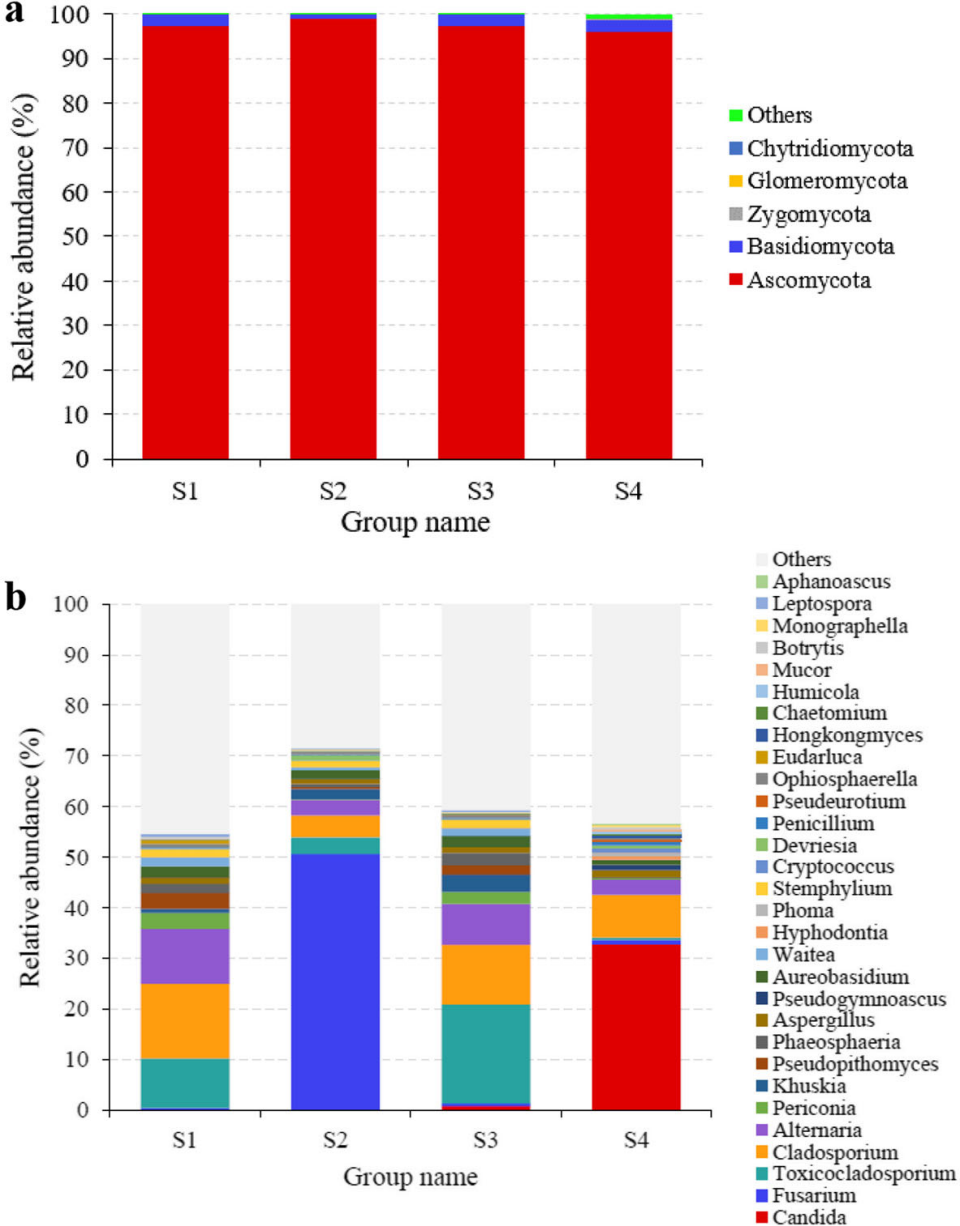
Relative abundances of fungal phyla (**a**) and genera (**b**) l in soil with different degree of deterioration from Jinsha earthen site. S1, no obvious deterioration; S2, mild deterioration; S3, moderate deterioration; S4, severe deterioration

The fungal communities were assigned into 5 phyla and 205 genera. In all sample groups, Ascomycota was the most abundant phylum and Basidiomycota the second most abundant with relative abundances ranging from 95.9 to 98.8% and 1.1 to 2.7%, respectively (Fig. 4a, Additional File 1: Table S4). At the genus level, the relative abundances of *Toxicocladosporium* and *Alternaria* were higher in sample groups S1 and S3 than in S2 and S4 (*p* < 0.05), that of *Fusarium* was highest in S2 (*p* < 0.05) and that of *Candida* in S4 (Fig. 4b, Additional File 1: Table S5).

Liner discriminant analysis (LDA) coupled with effect size (LEfSE) was used to identify differentially abundant taxa. Forty-six bacterial taxa were differentially abundant among the four sample groups. Three taxa were significantly more abundant in S1 than in the other three sample groups, six taxa in S2, sixteen taxa in S3, and 21 taxa in S4 (Additional File 1: Figure S2a). Thirty fungal taxa were differentially abundant. Fifteen taxa were significantly more abundant in S1 than in the other three sample groups, ten taxa in S3, and five taxa in S4 (Additional File 1: Figure S2b). The LDA scores of bacterial phylum Bacteroidetes and genus *Bacteroides*, and that of fungal class Saccharomycetes that includes *Candida* sp. were approximately five in the S4 samples, suggesting that these organisms were specific for the most deteriorated samples.

### The correlation between the microbial community and environmental factors

**Fig. 5:**
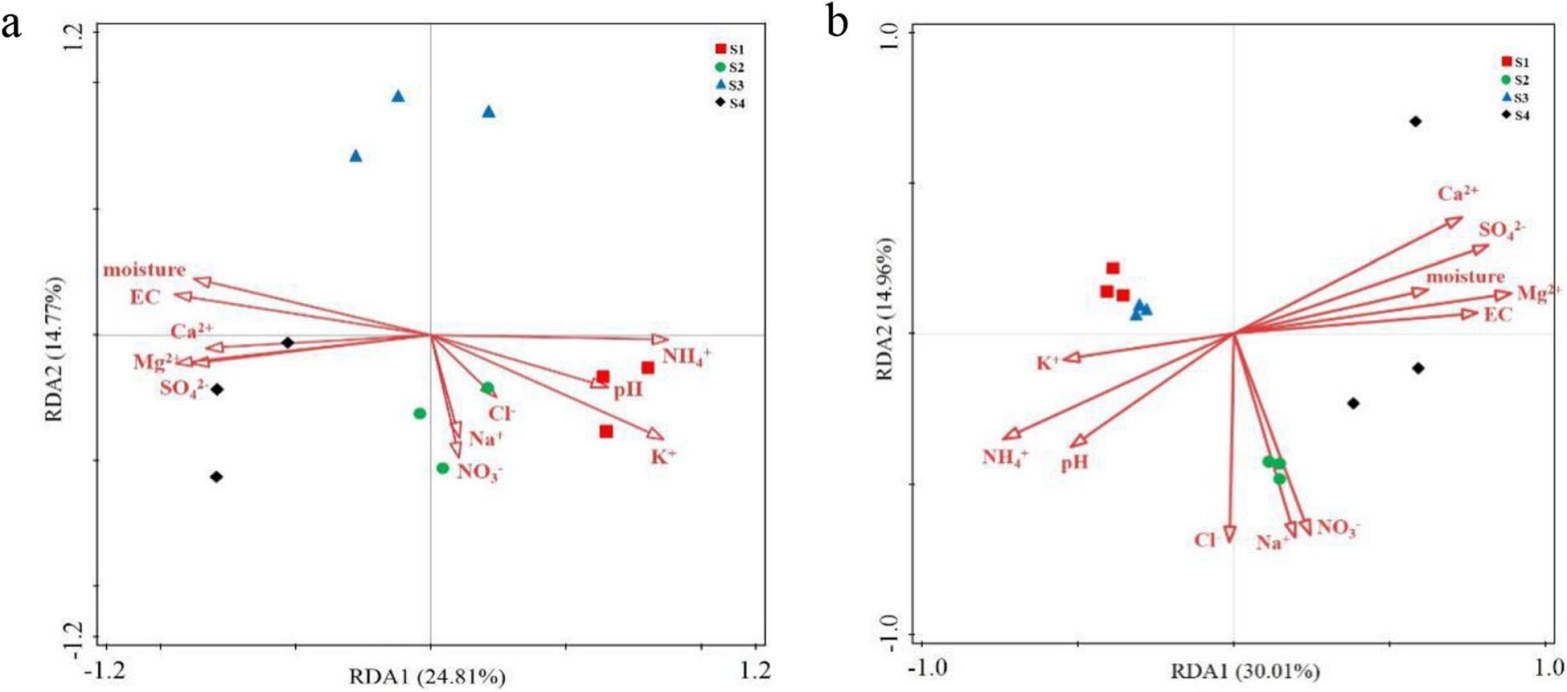
The relationships of environmental factors with bacterial (**a**) and fungal (**b**) communities in soil with different degree of deterioration from Jinsha earthen site. S1, no obvious deterioration; S2, mild deterioration; S3, moderate deterioration; S4, severe deterioration

The relationship between community compositions and environmental factors was analyzed using redundancy analysis (RDA). For bacteria, the RDA axes 1 and 2 accounted for 24.8 and 14.8%, respectively, of the total variation (Fig. 5a); for fungi, 30.0 and 15.0%, respectively (Fig. 5b). For both bacteria and fungi, the differences in community composition were associated with differences in EC, moisture, pH, and the concentrations of NH_4_^+^, K^+^, Mg^2+^, Ca^2+^ and SO_4_^2−^.

## Discussion

In this study, we analyzed bacterial and fungal community composition in soils from an earthen wall with different degree of deterioration at Jinsha earthen site, Chengdu, China, using high-throughput sequencing approach. For bacteria, the relative abundance of Actinobacteria was four times lower in the moderately and severely deteriorated sample groups S3 and S4 than in the not or mildly deteriorated sample groups S1 and S2; those of Proteobacteria and Firmicutes were highest in S3 and S4. However, it should be noted that changes in absolute abundances cannot be concluded from the relative abundance data [12]. Actinobacteria have been frequently found as a dominant group in subterranean micro-niches, including cultural relics in caves and wall paintings in catacombs [13–17]. Previous work demonstrated that some Actinobacteria are potentially harmful to the preservation of cultural relics [18–20]. Proteobacteria are commonly the most abundant bacteria in soil [21, 22], and Firmicutes that are tolerant to extreme temperatures and low humidity are often found in extreme environments [17]. These bacteria may play an important role in the microecological balance of earthen sites.

Among the bacterial genera, the relative abundances of *Rubrobacter* were highest in all but the most deteriorated sample group. *Rubrobacter* was considered connected with the biodeterioration of cultural relics and the rosy discoloration of masonry and lime wall paintings in historical buildings in Austria and Germany [23]. Resistance to desiccation might be a selective advantage for *Rubrobacter* growth, and efflorescence on walls might be due to *Rubrobacter* strains [24]. Therefore, *Rubrobacter* may play a crucial role at the early stage of deterioration of earthen sites. However, in the moderately and severely deteriorated sample groups S3 and S4, the relative abundances of *Rubrobacter* were remarkably lower than in S1 and S2. *Bacteroides* were specific for the most deteriorated samples. *Bacteroides* have the capability to produce acid [25], and the acid produced may dissolve minerals and further damage earthen features.

The distribution of the fungal genera in the four sample groups seemed more random than that of bacteria. The fungal communities in S1 and S3 were similar, indicating that the fungal composition varied only little with the deterioration degree. Among the fungal genera, the relative abundances of the filamentous fungi *Cladosporium*, *Fusarium* and *Toxicocladosporium* were highest in the sample groups S1, S2 and S3, respectively. These genera are widely distributed in wall paintings in caves, catacombs and churches and have been isolated from severely decayed areas of stone artwork [15, 26, 27]. *Cladosporium* and *Fusarium* have been reported to produce extracellular enzymes and abundant mycelia that contribute to the mineral dissolution and mechanical destruction of soil structure [15, 26, 27]. The relative abundance of yeast *Candida* was highest in the severely deteriorated sample group S4, and Saccharomycetes that includes *Candida* was specific for the most deteriorated samples. *Candida* species have the ability to secrete extracellular metabolites and to acidify soil [28]. Potentially, the accumulation of *Candida* plays an important role in the deterioration of earthen features. Further work is needed to conclude whether controlling the growth of the bacteria and fungi with high relative abundances in the deteriorated samples can be applied to alleviate deterioration, especially since information on *Candida* species in cultural relics is scarce.

Environmental factors affect the diversity and distribution of microorganisms in soil. The microbial community structure can rapidly change in response to altered environmental conditions [29, 30]. In our study, the differences in microbial communities were associated with differences in moisture, pH, EC and concentrations of soluble salt ions. Moisture has been found a major factor in affecting microbial communities and their activities [31, 32]. The higher moisture in the moderately and severely deteriorated sample groups S3 and S4 was associated with higher microbial diversity. Soil pH is another major factor connected with soil microbial community structure. In our study, the pH was higher in the moderately and severely deteriorated sample groups S3 and S4 than in the not or mildly deteriorated sample groups S1 and S2. Bacterial communities have been found more sensitive to changes in pH than fungal communities [33]. This could partly explain the more pronounced difference in bacterial communities than in fungal communities along the difference in deterioration degree.

EC is apparently associated with soil salinity [34]. As in an earlier study [35], the relative abundance of Bacteroidetes was found to correlate positively with EC. Soluble salts are considered to cause damage on earthen sites [5, 8]. We found that the differences in microbial communities were associated with differences in soluble salt concentrations. The bacterial and fungal communities in samples with no obvious deterioration correlated positively with K^+^ concentration, and those in severely deteriorated samples with Mg^2+^, Ca^2+^ and SO_4_^2−^ concentrations. Since the deterioration of earthen features releases nutrients [36], the nutrients released may have influenced the microbial diversity. Minerals are the main component of soil, the primary constituent of earthen features, accounting for more than 90% of the total solid phase of soil [37]. The dissolution of minerals could cause deterioration of earthen features by destroying soil structure and reducing stability. Plausibly, the dissolution of the Jinsha site mineral components lead to higher concentrations of Mg^2+^ and Ca^2+^ in the moderately and severely deteriorated sample groups S3 and S4. The nutrients released by soil mineral dissolution could support the growth and metabolism of microorganisms. As the growth and metabolism of microorganisms results in damage for cultural heritage relics [38], the preservation of earthen features may potentially benefit from preventing increases in diversity and possibly more active metabolism.

## Conclusions

In this study, we applied high throughput sequencing to explore microbial community structures in earthen wall with different degree of deterioration from Jinsha earthen site. The bacterial communities varied more than the fungal communities along the difference in deterioration degree. The differences in microbial community composition were associated with differences in soil physicochemical properties. Microbial diversity, soil moisture and the concentrations of Mg^2+^, Ca^2+^ and SO_4_^2−^ were higher and soil pH was lower with the increased degree of earthen wall deterioration. The results may potentially benefit the preservation of earthen sites.

## Methods

### Sampling

**Fig. 6:**
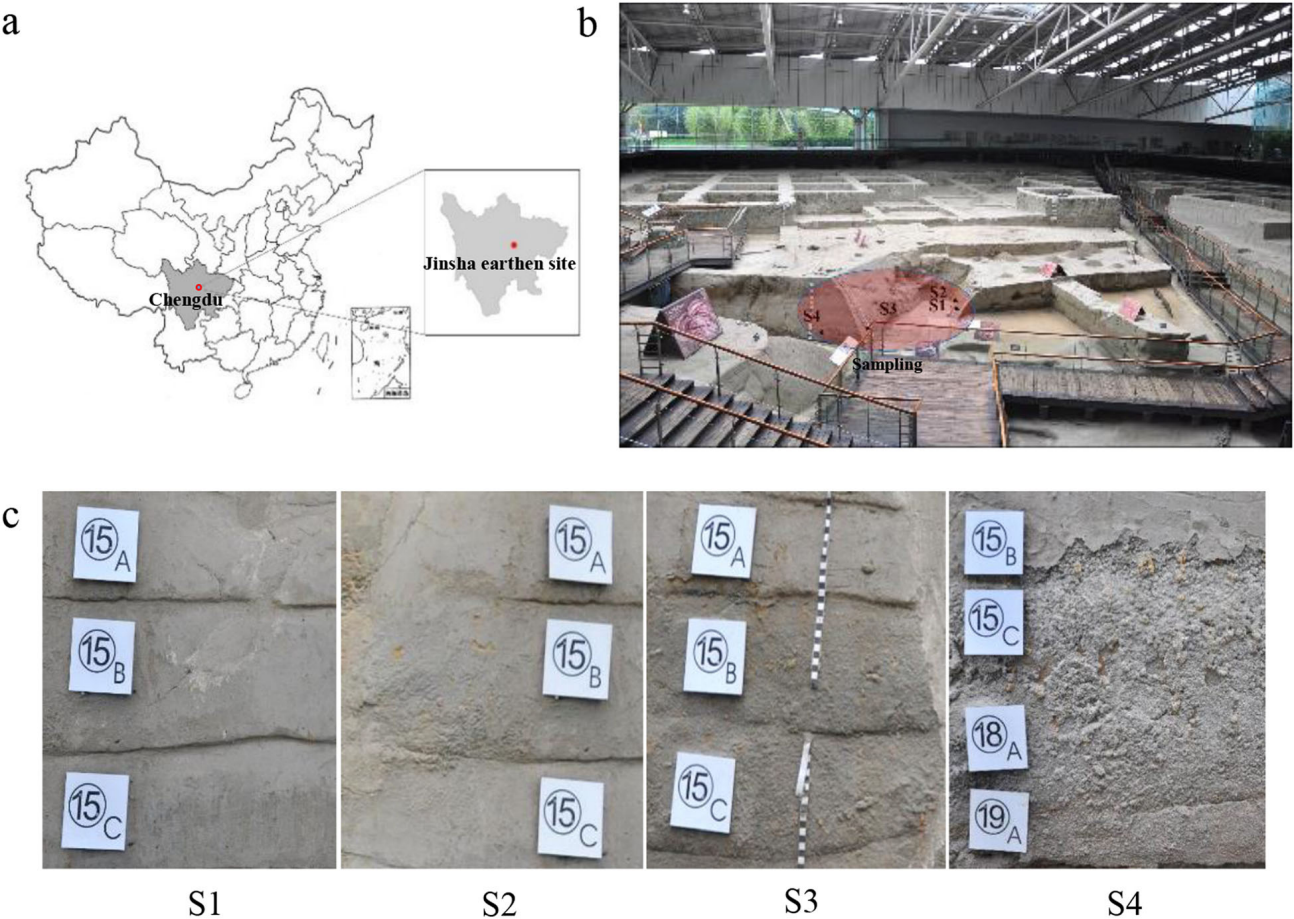
The locations of Jinsha site museum in Chengdu, Sichuan, China (**a**), sampling sites in the Relics Hall at Jinsha earthen site (**b**), and the individual samples (**c**). S1, no obvious deterioration; S2, mild deterioration; S3, moderate deterioration; S4, severe deterioration

Jinsha Site Museum is at No.2 Jinsha Site Road (30.68091 N, 104.01362 E), Chengdu, Sichuan, China (Fig. 6a). The earthen wall of the Ivory pit, formed during excavating in a sacrificial area with a lot of buried ivory, had undergone different degree of deterioration. Triplicate soil samples were randomly taken from the same cultural deposit layer (15C) in the Ivory pit wall with no obvious, mild, moderate and severe deterioration, and referred to as S1, S2, S3 and S4 sample groups, respectively (Fig. 6b, Fig. 6c). Samples were taken using minimally invasive sampling techniques and aseptic procedures, and transported on ice to the laboratory.

### Physicochemical properties analyses

Due to the minimal intervention principle in sampling in an archaeological site, the sample quantities were too low to meet the requirements of routine soil property analyses. We analyzed pH, moisture, electrical conductivity (EC) and soluble salt contents that are considered to play significant roles in the deterioration of earthen sites [1, 3, 8, 39]. The samples were air-dried, crushed and sieved through a ø 1 mm sieve. The determination of pH and EC were done in a 1:1 slurry of air-dried soil and water [40]. Moisture was determined by the oven drying method [41]. Soluble salt contents were measured using ion chromatography (IC): 2 g sample was suspended into 20 mL deionized water, the suspension was shaken for 30 min at 150 rpm, filtered first through medium pore sized filter paper and finally through 0.2 μm pore size syringe membrane filter [42]. Ions in the extracts were determined using a Dionex ICS-3000 ion chromatography system with an anion suppressor, a cation suppressor and a conductometric detector (Dionex Corporation, Sunnyvale, USA). Anions and cations in 25 μL of extract were analyzed using 4 mm × 50 mm guard columns AG11-11C and CG12A, respectively, 4 mm × 250 mm analytical columns IonPac AS11-HC and IonPac CS12A, respectively, and 20 mmol L^− 1^ sodium hydroxide solution and methanesulfonic acid solution, respectively, at 1 mL min^− 1^. The main elements in the samples were analyzed using scanning electron microscope EVO18 and energy dispersive spectrometer X-Max^N^ (SEM-EDS) (Carl Zeiss, Jena, Germany). The main minerals at Jinsha earthen site are quartz, feldspar, illite, montmorillonite and chlorite, with SiO_2_, Al_2_O_3_, Fe_2_O_3_, K_2_O, MgO, and CaO as the main chemical components (Additional File 1: Table S6) [43].

### DNA extraction

DNA was extracted from 0.5 g fresh sample using Fast DNA® SPIN for Soil Kit (MP BIO Laboratories, California, USA) according to the manufacturer’s instructions. The concentration and purity of DNA were checked by electrophoresis in 1.0% agarose gel and NanoDrop spectrophotometer (Thermo Scientific Inc., USA). DNA samples were stored at − 20 °C.

### 16S rRNA and ITS amplicon sequencing

The DNA samples were sequenced at Novogene Bioinformatics Technology, Co., Ltd. (Beijing, China). The V3-V4 regions of 16S rRNA genes were amplified using the primers 341F (5′-CCT AYG GGR BGC ASC AG-3′) and 806R (5′-GG ACT ACN NGG GTA TCT AAT-3′) [44]. The ITS2 region of ITS was amplified using the primers ITS3-2024F (5′-GCA TCG ATG AAG AAC GCA GC-3′) and ITS4-2409R (5′-TCC TCC GCT TAT TGA TAT GC-3′) [45]. The primers included sequencing specific adaptor sequences. Amplification was done in 30 μL reactions with 15 μL of Phusion®High-Fidelity PCR Master Mix (New England Biolabs), 0.2 μM of forward and reverse primers, and approximately 10 ng of template DNA. Thermal cycling consisted of initial denaturation at 98 °C for 1 min, followed by 30 cycles of denaturation at 98 °C for 10 s, annealing at 50 °C for 30 s, and elongation at 72 °C for 30 s, and a final elongation at 72 °C for 5 min and cooling at 4 °C.

Amplification was checked by mixing equal volume of 1X loading buffer with SYBR green and PCR product, and subjecting the mixture to electrophoresis in 2% agarose gel. A bright band at 400–500 bp indicated successful amplification. PCR products were purified with GeneJET Gel Extraction Kit (Thermo Scientific). Ion Plus Fragment Library Kit 48 rxns (Thermo Scientific) was used to generate sequencing libraries following manufacturer’s recommendations. The quality of the libraries was assessed on the Qubit 2.0 Fluorometer (Thermo Scientific). Finally, the libraries were sequenced on a Thermo Fisher Scientific Ion S5 XL platform and 600 bp single-end reads were generated.

### Bioinformatics and statistical analyses

Single-end reads were assigned to samples according to their unique barcodes and primers and barcodes were cut off. Low-quality sequences and reads with ambiguous nucleotides were removed using Cutadapt V1.9.1 [46]. Chimeric reads and sequences with ambiguous bases and average base quality score < 30 were filtered out using UCHIME v. 4.2.4.0 [47]. Sequences were assigned to operational taxonomic units (OTUs) at ≥97% similarity using UPARSE v7.0.1001 [48]. The representative sequences of the 16S rRNA OTUs were assigned to taxa using Silva 132 database and Mothur v1.36.1 [49, 50]. The representative sequences of the ITS OTUs were assigned to taxa using Unite database v7.2 (https://unite.ut.ee/) and QIIME v1.9.1 [51]. Chao1 and Shannon indices were calculated using the phyloseq package [52]. Venn diagrams were done at VennDiagramWeb [53]. The differences in microbial community composition between samples were visualized using principal component analysis (PCA) in CANOCO 5 [54]. Differential taxa at phylum to species levels were identified using linear discriminant analysis coupled with effect size (LEfSe) [55]. To analyze the bacterial and fungal community distribution and their correlation with environmental factors, redundancy analysis (RDA) was carried out using CANOCO 5 [54]. Statistical differences among groups were analyzed using one-way ANOVA followed by a post hoc least significance difference test (SPSS 17.0) [56]. Differences were taken statistically significant at *p* < 0.05.

The amplicon sequencing data were deposited into the NCBI Sequence Read Archive (SRA) under the accession numbers SRR9678166-SRR9678177 and SRR9678184-SRR9678195.

## Additional File

**Additional file 1 **: **Table S1** Elemental composition of soil with different degree of deterioration from Jinsha earthen site as determined by Scanning electron microscope - energy dispersive spectrometer (SEM-EDS). **Table S2** The proportions of ten most abundant bacterial phyla in soil with different degree of deterioration from Jinsha earthen site. S1, no obvious deterioration; S2, mild deterioration; S3, moderate deterioration; S4, severe deterioration. **Table S3** The proportions of ten most abundant bacterial genera in soil with different degree of deterioration from Jinsha earthen site. S1, no obvious deterioration; S2, mild deterioration; S3, moderate deterioration; S4, severe deterioration. **Table S4** The proportions of fungal phyla in soil with different degree of deterioration from Jinsha earthen site. S1, no obvious deterioration; S2, mild deterioration; S3, moderate deterioration; S4, severe deterioration. **Table S5** The proportions of thirty most abundant fungal genera in soil with different degree of deterioration from Jinsha earthen site. S1, no obvious deterioration; S2, mild deterioration; S3, moderate deterioration; S4, severe deterioration. **Table S6** The mineral and chemical components at Jinsha earthen site, Chengdu, China. Data from Li (2007). **Figure S1** The rarefaction curves of bacterial (a) and fungal (b) operational taxonomic units (OTUs) in soil with different degree of deterioration from Jinsha earthen site. **Figure S2** Differentially abundant bacterial (a) and fungal (b) taxa in soil with different degree of deterioration from Jinsha earthen site. S1, no obvious deterioration; S2, mild deterioration; S3, moderate deterioration; S4, severe deterioration.

## Data Availability

The raw sequence data on 16S rRNA gene and ITS amplicons have been submitted to the NCBI Sequence Read Archive (SRA) database with the accession numbers SRR9678166-SRR9678177 and SRR9678184-SRR9678195.

## Abbreviations

16S rRNA: 16S ribosomal RNA
ITS: Internal transcribed spacer
OTU: Operational taxonomic unit
PCA: Principal component analysis
RDA: Redundancy analysis
One-way ANOVA: One-way analysis of variance
LEfSe: Linear discriminant analysis coupled with effect size
LDA: Liner discriminant analysis
SEM-EDS: Scanning electron microscope - energy dispersive spectrometer
EC: Electrical conductivity
IC: Ion chromatography

## References

[1] Luo X, Gu Z, Yu C (2015). Desiccation cracking of earthen sites in archaeology museum - a viewpoint of chemical potential difference of water content. Indoor Built Environ.

[2] Li Z, Wang X, Sun M, Chen W, Guo Q, Zhang H (2011). Conservation of Jiaohe ancient earthen site in China. J Rock Mech Geotech Eng.

[3] Li L, Shao M, Wang S, Li Z (2011). Preservation of earthen heritage sites on the silk road, Northwest China from the impact of the environment. Environ Earth Sci.

[4] Shao M, Li L, Wang S, Wang E, Li Z (2013). Deterioration mechanisms of building materials of Jiaohe ruins in China. J Cult Herit.

[5] Zhang Y, Ye WM, Chen B, Chen YG, Ye B (2016). Desiccation of NaCl-contaminated soil of earthen heritages in the site of Yar City, Northwest China. Appl Clay Sci.

[6] Du Y, Chen W, Kai C, Gong S, Pu T, Fu X (2017). A model characterizing deterioration at earthen sites of the Ming Great Wall in Qinghai province, China. Soil Mech Found Eng.

[7] Rolón G, Cilla G (2012). Adobe wall biodeterioration by the Centris muralis Burmeister bee (Insecta: Hymenoptera: Apidae) in a valuable colonial site, the Capayán ruins. Int Biodeterior Biodegradation.

[8] Qian L, Xia Y, Hu H, Zhang S, Lv G, Chen G (2017). A study on the soluble salt contents and occurrence state in the soil of the earthen site at Xiongjiazong. Dunhuang Res.

[9] Yukiyasu F, Enrico F, Kunio W, Kazuya M (2009). Digital photogrammetry for the documentation of structural damage in earthen archaeological sites: the case of Ajina Tepa, Tajikistan. Eng Geol.

[10] Chen Y (2018). Study of temperature effect induced by insolation on deterioration of earthen monument in arid areas.

[11] Zhang G, Gong C, Gu J, Kateyama Y, Someya T, Gu J-D (2019). Biochemical reactions and mechanisms involved in the biodeterioration of stone world cultural heritage under the tropical climate conditions. Int Biodeterior Biodegradation.

[12] Gloor GB, Macklaim JM, Pawlowsky-Glahn V, Egozcue JJ (2017). Microbiome datasets are compositional: and this is not optional. Front Microbiol.

[13] Stomeo F, Portillo MC, Gonzalez JM, Laiz L, Saiz-Jimenez C (2008). Pseudonocardia in white colonizations in two caves with Paleolithic paintings. Int Biodeterior Biodegrad.

[14] Jurado V, Laiz L, Rodriguez-Nava V, Boiron P, Hermosin B, Sanchez-Moral S (2010). Pathogenic and opportunistic microorganisms in caves. Int J Speleol.

[15] Qiang L, Zhang B, Wang L, Ge Q (2017). Distribution and diversity of bacteria and fungi colonizing ancient Buddhist statues analyzed by high-throughput sequencing. Int Biodeterior Biodegrad.

[16] Duan Y, Wu F, Wang W, He D, Gu JD, Feng H (2017). The microbial community characteristics of ancient painted sculptures in Maijishan grottoes, China. PLoS One.

[17] Duan Y, Wu F, Wang W, Gu JD, Li Y, Feng H (2018). Differences of microbial community on the wall paintings preserved in situ and ex situ of the Tiantishan grottoes, China. Int Biodeterior Biodegrad.

[18] Beata G, Sukriye C-A, Vincent B, Anna O, Egemen A, Athenia LO (2015). Metabolomic and high-throughput sequencing analysis-modern approach for the assessment of biodeterioration of materials from historic buildings. Front Microbiol.

[19] Diazherraiz M, Jurado V, Cuezva S, Laiz L, Pallecchi P, Tiano P (2013). The Actinobacterial colonization of Etruscan paintings. Sci Rep.

[20] López-Miras M (2013). Microbial communities adhering to the obverse and reverse sides of an oil painting on canvas: identification and evaluation of their biodegradative potential. Aerobiologia..

[21] Li Z, Xu Z (2008). Assessing bacterial diversity in soil. J Soils Sediments.

[22] Spain AM, Krumholz LR, Elshahed MS (2009). Abundance, composition, diversity and novelty of soil Proteobacteria. ISME J.

[23] Schabereiter-Gurtner C, Piñar G, Vybiral D, Lubitz W, Rölleke S (2001). *Rubrobacter* -related bacteria associated with rosy discolouration of masonry and lime wall paintings. Arch Microbiol.

[24] Laiz L, Miller AZ, Jurado V, Akatova E, Sanchez-Moral S, Gonzalez JM (2009). Isolation of five *Rubrobacter* strains from biodeteriorated monuments. Naturwissenschaften..

[25] Maki K, Mitsuo S, Masako I, Shinji S, Yoshimi B (2005). *Bacteroides plebeius* sp. nov. and *Bacteroides coprocola* sp. nov., isolated from human faeces. Int J Syst Evol Microbiol.

[26] Wang W, Wang Y, Wang H, Ma X, Yi W (2016). Effects of different continuous cropping and rotation of poplar plantation on soil nitrogen Bacteria community and nitrogen metabolism. Sci Silvae Sin.

[27] Dupont J, Jacquet C, Dennetière B, Lacoste S, Bousta F, Orial G (2007). Invasion of the French paleolithic painted cave of Lascaux by members of the *Fusarium solani* species complex. Mycologia..

[28] Botha A (2011). The importance and ecology of yeasts in soil. Soil Biol Biochem.

[29] Fierer N, Schimel JP, Holden PA (2003). Variations in microbial community composition through two soil depth profiles. Soil Biol Biochem.

[30] Gu Y, Wang Y, Lu S, Xiang Q, Yu X, Zhao K (2017). Long-term fertilization structures bacterial and archaeal communities along soil depth gradient in a paddy soil. Front Microbiol.

[31] Zumsteg A, Bååth E, Stierli B, Zeyer J, Frey B (2013). Bacterial and fungal community responses to reciprocal soil transfer along a temperature and soil moisture gradient in a glacier forefield. Soil Biol Biochem.

[32] Bell C, Mcintyre N, Cox S, Tissue D, Zak J (2008). Soil microbial responses to temporal variations of moisture and temperature in a Chihuahuan Desert grassland. Microb Ecol.

[33] Rousk J, Baath E, Brookes PC, Lauber CL, Lozupone C, Caporaso JG (2010). Soil bacterial and fungal communities across a pH gradient in an arable soil. ISME J.

[34] Hardie M, Doyle R (2012). Measuring soil salinity. Methods Mol Biol.

[35] Kim JM, Roh AS, Choi SC, Kim EJ, Choi MT, Ahn BK (2016). Soil pH and electrical conductivity are key edaphic factors shaping bacterial communities of greenhouse soils in Korea. J Microbiol.

[36] Uroz S, Calvaruso C, Turpault MP, Pierrat JC, Mustin C, Frey-Klett P (2007). Effect of the mycorrhizosphere on the genotypic and metabolic diversity of the bacterial communities involved in mineral weathering in a forest soil. Appl Environ Microbiol.

[37] Bai J, Chao Y, Chen Y, Wang S, Qiu R. The effect of interaction between *Bacillus subtilis* DBM and soil minerals on cu (II) and Pb (II) adsorption [J]. J Environ Sci. 2018:328–37. 10.1016/j.jes.2018.11.012. 10.1016/j.jes.2018.11.012 30665652

[38] Raschle P (2001). Microbiology for our cultural heritage. Chimia Int J Chem.

[39] Hu H, Xia Y, Jin Z, Zhang S, Rong B, Wang L (2012). Study on the salt species and types in the emperor Qin's mausoleum and Hanyangling mausoleum earthen sites. Materials China.

[40] Smith JL, John WD. [SSSA Special Publication] Methods for assessing soil quality || measurement and use of pH and electrical conductivity for soil quality analysis. Methods Assessing Soil Quanl. 1996:169–85 https://www.onacademic.com/detail/journal_1000040019195510_ccca.html.

[41] Schmugge TJ, Jackson TJ, Mckim HL (1980). Survey of methods for soil moisture determination. Water Resour Res.

[42] Stanišić SM, Manojlović DD, Dojčinović BP (2011). The comparison of sample extraction procedures for the determination of cations in soil by IC and ICP-AES. Cent Eur J Chem.

[43] Li X (2007). Preparation, performance study and application of a new consolidation material for earthen archaeological site.

[44] Yu Y, Lee C, Kim J, Hwang S (2005). Group-specific primer and probe sets to detect methanogenic communities using quantitative real-time polymerase chain reaction. Biotechnol Bioeng.

[45] Lott TJ, Kuykendall RJ, Reiss E (1993). Nucleotide sequence analysis of the 5.8S rDNA and adjacent ITS2 region of Candida albicans and related species. Yeast..

[46] Martin M (2011). Cutadapt removes adapter sequences from high-throughput sequencing reads. Embnet J.

[47] Edgar RC, Haas BJ, Clemente JC, Christopher Q, Rob K (2011). UCHIME improves sensitivity and speed of chimera detection. Bioinformatics..

[48] Edgar RC (2013). UPARSE: highly accurate OTU sequences from microbial amplicon reads. Nat Methods.

[49] Christian Q, Elmar P, Pelin Y, Jan G, Timmy S, Pablo Y (2012). The SILVA ribosomal RNA gene database project: improved data processing and web-based tools. Nucleic Acids Res.

[50] Wang Q, Garrity GM, Tiedje JM, Cole JR (2007). Naive Bayesian classifier for rapid assignment of rRNA sequences into the new bacterial taxonomy. Appl Environ Microbiol.

[51] Caporaso JG, Kuczynski J, Stombaugh J, Bittinger K (2010). QIIME allows analysis of high-throughput community sequencing data. Nat Methods.

[52] Langfelder P, Horvath S (2008). WGCNA: an R package for weighted correlation network analysis. BMC Bioinformatics.

[53] Lam F, Lalansingh CM, Babaran HE, Wang Z, Prokopec SD, Fox NS (2016). VennDiagramWeb: a web application for the generation of highly customizable Venn and Euler diagrams. BMC Bioinformatics.

[54] Šmilauer P, Lepš J (2014). Multivariate analysis of ecological data using CANOCO 5.

[55] Segata N, Izard J, Waldron L, Gevers D, Miropolsky L, Garrett WS (2011). Metagenomic biomarker discovery and explanation. Genome Biol.

[56] Stern L (2009). A Visual Approach to SPSS for Windows: A Guide to SPSS 17.0: Allyn and Bacon, Inc.

