# Supplementary material for: Changes in soil microbial communities at Jinsha earthen site are associated with earthen site deterioration
Source: BMC Microbiol. 2020 Jun 5;20:147. doi: 10.1186/s12866-020-01836-1 (PMC7275329; doi:10.1186/s12866-020-01836-1)
Supplement: Supplementary file 1 — Additional file 1 : Table S1 Elemental composition of soil with different degree of deterioration from Jinsha earthen site as determined by Scanning electron microscope - energy dispersive spectrometer (SEM-EDS). Table S2 The proportions of ten most abundant bacterial phyla in soil with different degree of deterioration from Jinsha earthen site. S1, no obvious deterioration; S2, mild deterioration; S3, moderate deterioration; S4, severe deterioration. Table S3 The proportions of ten most abundant bacterial genera in soil with different degree of deterioration from Jinsha earthen site. S1, no obvious deterioration; S2, mild deterioration; S3, moderate deterioration; S4, severe deterioration. Table S4 The proportions of fungal phyla in soil with different degree of deterioration from Jinsha earthen site. S1, no obvious deterioration; S2, mild deterioration; S3, moderate deterioration; S4, severe deterioration. Table S5 The proportions of thirty most abundant fungal genera in soil with different degree of deterioration from Jinsha earthen site. S1, no obvious deterioration; S2, mild deterioration; S3, moderate deterioration; S4, severe deterioration. Table S6 The mineral and chemical components at Jinsha earthen site, Chengdu, China. Data from Li (2007). Figure S1 The rarefaction curves of bacterial (a) and fungal (b) operational taxonomic units (OTUs) in soil with different degree of deterioration from Jinsha earthen site. Figure S2 Differentially abundant bacterial (a) and fungal (b) taxa in soil with different degree of deterioration from Jinsha earthen site. S1, no obvious deterioration; S2, mild deterioration; S3, moderate deterioration; S4, severe deterioration. [file 12866_2020_1836_MOESM1_ESM.docx]

**Supplementary Table Titles**

**Table S1** Elemental composition of soil with different degree of deterioration from Jinsha earthen site as determined by Scanning electron microscope - energy dispersive spectrometer (SEM-EDS).

**Table S2** The proportions of ten most abundant bacterial phyla in soil with different degree of deterioration from Jinsha earthen site. S1, no obvious deterioration; S2, mild deterioration; S3, moderate deterioration; S4, severe deterioration.

**Table S3** The proportions of ten most abundant bacterial genera in soil with different degree of deterioration from Jinsha earthen site. S1, no obvious deterioration; S2, mild deterioration; S3, moderate deterioration; S4, severe deterioration.

**Table S4** The proportions of fungal phyla in soil with different degree of deterioration from Jinsha earthen site. S1, no obvious deterioration; S2, mild deterioration; S3, moderate deterioration; S4, severe deterioration.

**Table S5** The proportions of thirty most abundant fungal genera in soil with different degree of deterioration from Jinsha earthen site. S1, no obvious deterioration; S2, mild deterioration; S3, moderate deterioration; S4, severe deterioration.

**Table S6** The mineral and chemical components in Jinsha earthen site, Chengdu, China. Data from Li (2007).

**Supplementary Figure Legends**

**Figure S1** The rarefaction curves of bacterial (a) and fungal (b) operational taxonomic units (OTUs).

**Figure S2** Differentially abundant bacterial (a) and fungal (b) taxa in soil with different degree of deterioration from Jinsha earthen site. S1, no obvious deterioration; S2, mild deterioration; S3, moderate deterioration; S4, severe deterioration.

**Table S1**

| Sample | Elements (w/%) | | | | | | | | | |
| --- | --- | --- | --- | --- | --- | --- | --- | --- | --- | --- |
|  | C | O | Na | Mg | Al | Si | S | K | Ti | Fe |
| S1 | 10.43±1.13^c^ | 32.15±1.36^b^ | 0.34±0.06^a^ | 0.86±0.10^c^ | 19.51±0.76^a^ | 24.27±0.32^a^ | - | 10.40±0.30^a^ | - | 1.21±0.21^bc^ |
| S2 | 12.46±0.84^b^ | 33.67±0.93^b^ | 0.55±0.09^a^ | 1.00±0.04^c^ | 18.33±0.78^b^ | 23.29±0.27^a^ | - | 8.39±0.30^b^ | 0.34±0.10^a^ | 1.98±0.36^b^ |
| S3 | 16.63±0.91^a^ | 44.61±0.71^a^ | 0.41±0.41^a^ | 8.49±0.36^b^ | 8.02±0.06^c^ | 9.57±0.83^b^ | 1.90±0.23^b^ | 0.01±0.01^d^ | - | 10.35±0.8^a^ |
| S4 | 16.81±0.72^a^ | 46.31±1.37^a^ | - | 13.86±0.78^a^ | 1.80±0.43^d^ | 2.11±1.34^c^ | 18.04±0.51^a^ | 0.55±0.08^c^ | - | 0.52±0.14^c^ |

The results are average ± standard deviation (n=3). Different superscript letters in a column indicate statistical significant difference (p < 0.05) in the least significant difference test. S1, no obvious deterioration; S2, mild deterioration; S3, moderate deterioration; S4, severe deterioration; –, not detected.

**Table S2**

| Taxonomy | Relative Abundance | | | |
| --- | --- | --- | --- | --- |
|  | S1 | S2 | S3 | S4 |
| Actinobacteria | 76.67%±2.54%^a^ | 82.18%±10.7%^a^ | 20.99%±9.78%^b^ | 18.03%±16.54%^b^ |
| Bacteroidetes | 0.93%±0.38%^a^ | 2.57%±1.99%^a^ | 16.02%±15.89%^a^ | 27.17%±23.09%^a^ |
| Proteobacteria | 8.83%±0.32%^b^ | 7.50%±3.57%^b^ | 33.91%±5.63%^a^ | 25.84%±7.86%^a^ |
| Firmicutes | 8.13%±0.87%^b^ | 5.55%±3.84%^b^ | 18.18%±4.92%^a^ | 23.63%±5.03%^a^ |
| Cyanobacteria | 4.09%±1.09%^a^ | 0.41%±0.21%^b^ | 3.58%±1.87%^a^ | 0.86%±1.19%^b^ |
| Fusobacteria | 0.04%±0.02%^a^ | 0.39%±0.33%^a^ | 0.97%±0.09%^a^ | 0.95%±0.70%^a^ |
| Acidobacteria | 0.15%±0.07%^a^ | 0.09%±0.09%^a^ | 0.47%±0.15%^a^ | 0.61%±0.57%^a^ |
| Deinococcus-Thermus | 0.46%±%0.13^ab^ | 0.75%±0.32%^a^ | 0.33%±0.24%^ab^ | 0.10%±0.14%^b^ |
| Chloroflexi | 0.10%±0.05%^a^ | 0.07%±0.05%^a^ | 0.39%±0.23%^a^ | 0.38%±0.43%^a^ |
| Gemmatimonadetes | 0.02%±0.03%^a^ | 0.04%±0.03%^a^ | 0.07%±0.06%^a^ | 0.22%±0.22%^a^ |
| Others | 0.58%±0.08%^a^ | 0.44%±0.37%^a^ | 5.09%±7.78%^a^ | 2.21%±2.24%^a^ |

The results are average ± standard deviation (n=3). Different superscript letters in a column indicate statistical significant difference (p < 0.05) in the least significant difference test. S1, no obvious deterioration; S2, mild deterioration; S3, moderate deterioration; S4, severe deterioration; –, not detected.

**Table S3**

| Taxonomy | Relative Abundance | | | |
| --- | --- | --- | --- | --- |
|  | S1 | S2 | S3 | S4 |
| Rubrobacter | 73.62%±2.74%^a^ | 74.43%±11.67%^a^ | 14.03%±6.22%^b^ | 0.75%±0.36%^b^ |
| Bacteroides | 0.15%±0.06%^a^ | 0.77%±0.68%^a^ | 10.33%±14.73%^a^ | 19.13%±23.05%^a^ |
| Corynebacterium_1 | 0.18%±0.06%^a^ | 0.13%±0.07%^a^ | 0.48%±0.38%^a^ | 7.9%±1.27%^a^ |
| Ralstonia | 0.25%±0.07%^a^ | 1.86%±0.17%^a^ | 7.31%±4.01%^a^ | 5.03%±4.68%^a^ |
| Faecalibacterium | 0.32%±0.31%^a^ | 1.05%±0.91%^a^ | 2.86%±2.62%^a^ | 4.56%±2.61%^a^ |
| Acinetobacter | 0.28%±0.12%^a^ | 0.26%±0.22%^a^ | 3.26%±2.44%^a^ | 0.89%±0.59%^a^ |
| Escherichia-Shigella | 0.02%±0.02%^a^ | 0.34%±0.23%^a^ | 1.23%±1.46%^a^ | 2.67%±2.75%^a^ |
| Propionibacterium | 0.38%±0.15%^a^ | 0.45%±0.25%^a^ | 1.43%±0.79%^a^ | 2.23%±2.89%^a^ |
| Shewanella | 0.21%±0.14%^b^ | 0.70%±0.62%^b^ | 2.82%±0.73%^a^ | 2.17%±0.28%^a^ |
| Halomonas | 0.30%±0.07%^b^ | 0.77%±0.65%^b^ | 2.49%±0.73%^a^ | 1.37%±0.69%^ab^ |
| Others | 24.54%±2.32%^a^ | 19.45%±8.63%^a^ | 55.83%±13.21%^a^ | 52.46%±19.01%^a^ |

The results are average ± standard deviation (n=3). Different superscript letters in a column indicate statistical significant difference (p < 0.05) in the least significant difference test. S1, no obvious deterioration; S2, mild deterioration; S3, moderate deterioration; S4, severe deterioration; –, not detected.

**Table S4**

| Taxonomy | Relative Abundance | | | |
| --- | --- | --- | --- | --- |
|  | S1 | S2 | S3 | S4 |
| Ascomycota | 97.24%±0.57%^a^ | 98.78%±1.30%^a^ | 97.35%±0.07%^a^ | 95.95%±2.7%^a^ |
| Basidiomycota | 2.75%±0.06%^a^ | 1.12%±1.11%^a^ | 2.42%±0.006%^a^ | 2.63%±1.68%^a^ |
| Zygomycota | <0.01% | 0.05%±0.08%^a^ | 0.12%±0.08%^a^ | 0.46%±0.63%^a^ |
| Glomeromycota | - | - | - | 0.02%±0.03%^a^ |
| Chytridiomycota | - | - | - | 0.01%±0.01%^a^ |
| Others | 0.01%±0.005%^b^ | 0.05%±0.09%^b^ | 0.11%±0.011%^b^ | 0.93%±0.81%^a^ |

The results are average ± standard deviation (n=3). Different superscript letters in a column indicate statistical significant difference (p < 0.05) in the least significant difference test. S1, no obvious deterioration; S2, mild deterioration; S3, moderate deterioration; S4, severe deterioration; –, not detected.

**Table S5**

| Taxonomy | Relative Abundance | | | |
| --- | --- | --- | --- | --- |
|  | S1 | S2 | S3 | S4 |
| Candida | 0.01%±0.00%^a^ | 0.12%±0.12%^a^ | 0.79%±0.35%^a^ | 32.74%±35.69%^a^ |
| Fusarium | 0.38%±0.36%^b^ | 50.56%±18.72%^a^ | 0.52%±0.19%^b^ | 0.93%±1.21%^b^ |
| Toxicocladosporium | 9.85%±1.02%^b^ | 3.31%±1.06%^c^ | 19.56%±3.06%^a^ | 0.4%±0.04%^c^ |
| Cladosporium | 14.78%±0.86%^a^ | 4.30%±2.90%^c^ | 11.76%±2.75%^ab^ | 8.45%±4.81%^bc^ |
| Alternaria | 10.78%±1.80%^a^ | 3.13%±0.61%^b^ | 8.17%±1.02%^a^ | 3.05%±3.10%^b^ |
| Periconia | 3.17%±0.54%^a^ | 0.12%±0.06%^c^ | 2.33%±0.43%^b^ | 0.23%±0.18%^c^ |
| Khuskia | 0.85%±0.15%^c^ | 1.9%±0.62%^b^ | 3.4%±0.25%^a^ | 0.05%±0.06%^d^ |
| Pseudopithomyces | 3.18%±0.42%^a^ | 0.38%±0.18%^c^ | 1.85%±0.40%^b^ | 0.05%±0.06%^c^ |
| Phaeosphaeria | 1.71%±0.36%^a^ | 0.55%±0.39%^b^ | 2.45%±0.84%^a^ | 0.16%±0.10%^b^ |
| Aspergillus | 1.3%±0.15%^a^ | 1.05%±0.29%^a^ | 1.18%±0.28%^a^ | 1.42%±1.27%^a^ |
| Pseudogymnoascus | - | 0.05%±0.09%^a^ | - | 0.97%±1.38%^a^ |
| Aureobasidium | 2.21%±0.19%^a^ | 1.65%±0.56%^ab^ | 2.22%±0.30%^a^ | 1.05%±0.71%^b^ |
| Waitea | 1.8%±0.47%^a^ | 0.59%±0.48%^b^ | 1.52%±0.60%^a^ | 0.01%±0.01%^b^ |
| Hyphodontia | 0.02%±0.02%^a^ | 0.02%±0.03%^a^ | 0.01%±0.01%^a^ | 0.69%±1.20%^a^ |
| Phoma | - | - | 0.02%±0.04%^a^ | 0.66%±1.13%^a^ |
| Stemphylium | 1.58%±0.27%^a^ | 1.31%±0.59%^a^ | 1.66%±0.23%^a^ | 0.02%±0.02%^b^ |
| Cryptococcus | 0.06%±0.01%^b^ | 0.05%±0.05%^b^ | 0.03%±0.03%^b^ | 0.86%±0.82%^a^ |
| Devriesia | 0.09%±0.02%^a^ | 1.03%±0.31%^a^ | 0.05%±0.01%^a^ | 0.6%±1.03%^a^ |
| Penicillium | 0.23%±0.13%^a^ | 0.20%±0.23%^a^ | 0.21%±0.12%^a^ | 0.69%±0.63%^a^ |
| Pseudeurotium | - | - | - | 0.44%±0.76%^a^ |
| Ophiosphaerella | 0.63%±0.21%^a^ | 0.46%±0.32%^a^ | 0.64%±0.06%^a^ | 0.44%±0.76%^a^ |
| Eudarluca | 0.83%±0.41%^a^ | 0.01%±0.00%^b^ | 0.03%±0.03%^b^ | - |
| Hongkongmyces | - | - | - | 0.42%±0.73%^a^ |
| Chaetomium | 0.10%±0.03%^a^ | 0.10%±0.12%^a^ | 0.19%±0.10%^a^ | 0.41%±0.70%^a^ |
| Humicola | - | - | - | 0.39%±0.67%^a^ |
| Mucor | - | - | - | 0.36%±0.62%^a^ |
| Botrytis | 0.41%±0.08%^a^ | 0.16%±0.23%^a^ | 0.24%±0.07%^a^ | 0.54%±0.44%^a^ |
| Monographella | 0.08%±0.02%^a^ | 0.18%±0.30%^a^ | 0.09%±0.08%^a^ | 0.33%±0.56%^a^ |
| Leptospora | 0.58%±0.17%^a^ | 0.24%±0.41%^a^ | 0.43%±0.42%^a^ | 0.01%±0.01%^a^ |
| Aphanoascus | - | - | - | 0.24%±0.42%^a^ |
| Others | 45.38%±1.62%^a^ | 28.54%±16.68%^a^ | 40.64%±25.67%^a^ | 43.37%±14.78%^a^ |

The results are average ± standard deviation (n=3). Different superscript letters in a column indicate statistical significant difference (p < 0.05) in the least significant difference test. S1, no obvious deterioration; S2, mild deterioration; S3, moderate deterioration; S4, severe deterioration; –, not detected.

**Table S6**

| Mineral component | Quartz | 20%-65% |
| --- | --- | --- |
|  | Feldspar | 9%-18% |
|  | Illite ilerite | 7%-30% |
|  | Montmorillonite | 2%-12% |
|  | Chlorite | 5%-18% |
|  | Clay minerals | 14%-60% |
| Chemical component | SiO_2_, Al_2_O_3_ | 60%-75% |
|  | Fe_2_O_3_, K_2_O, MgO, CaO | 10%-15% |
|  | Na_2_O, TiO_2_, P_2_O_5_, MnO | 1%-2% |

**Figure S1**


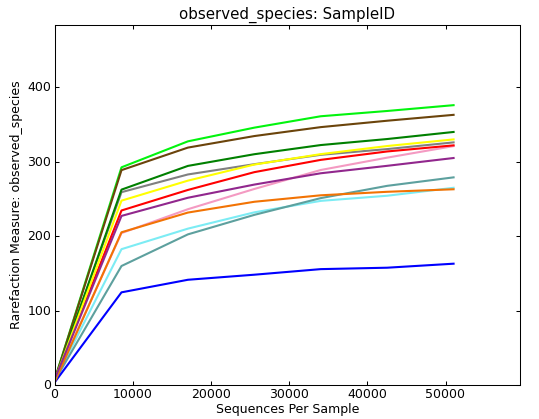

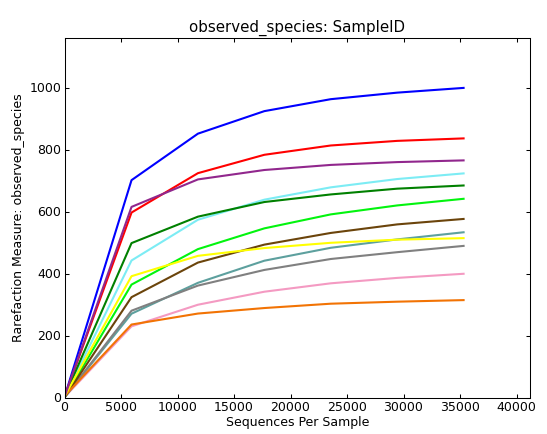


a

b

**Figure S2**


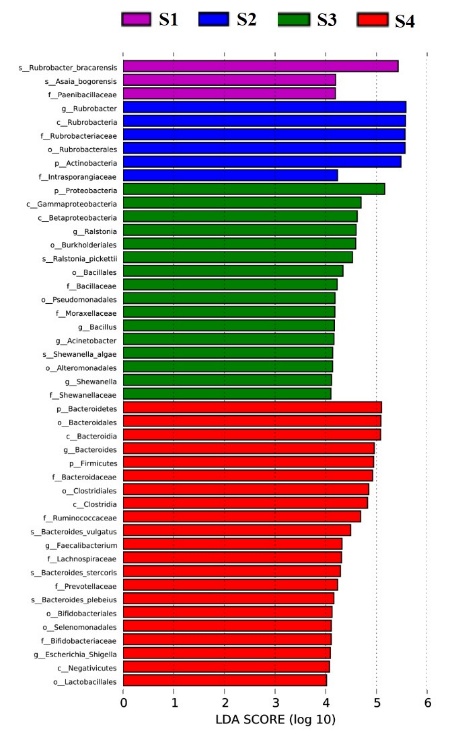

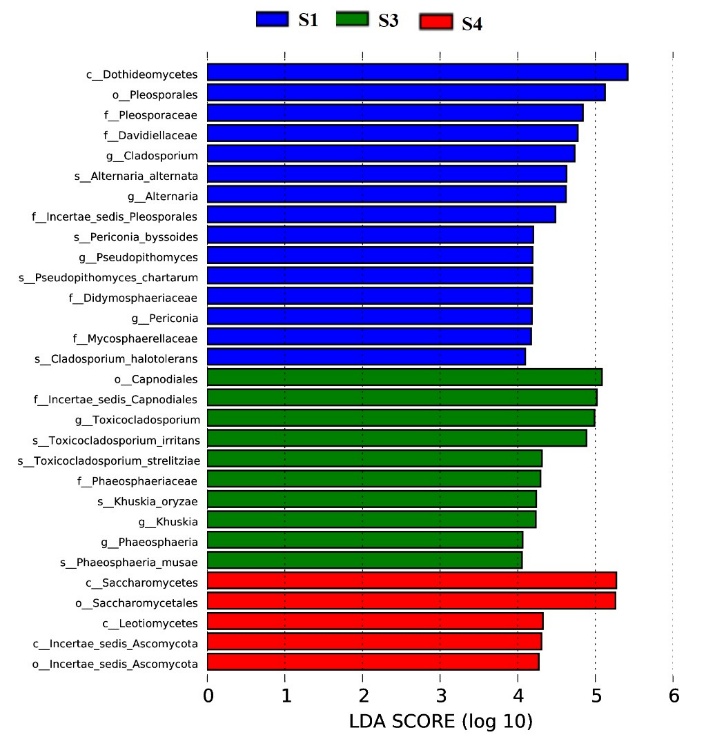


b

a
